# Impact of oral administration of single strain *Lactococcus lactis* spp. *cremoris* on immune responses to keyhole limpet hemocyanin immunization and gut microbiota: A randomized placebo-controlled trial in healthy volunteers

**DOI:** 10.3389/fimmu.2022.1009304

**Published:** 2022-12-07

**Authors:** Mahdi Saghari, Pim Gal, Hendrika W. Grievink, Erica S. Klaassen, Andrea Itano, Duncan McHale, Matthijs Moerland

**Affiliations:** ^1^ Centre for Human Drug Research (CHDR), Leiden, Netherlands; ^2^ Leiden University Medical Centre (LUMC), Leiden, Netherlands; ^3^ Leiden Academic Centre for Drug Research (LACDR), Leiden, Netherlands; ^4^ Evelo Biosciences Inc., Cambridge, MA, United States

**Keywords:** EDP1066, *Lactococcus lactis* spp. *cremoris*, gastrointestinal microbiome, keyhole limpet hemocyanin, late-phase skin reaction, delayed-type hypersensitivity, autoimmune disease

## Abstract

**Introduction:**

*Lactococcus lactis* spp. *cremoris* has been associated with promising immunomodulatory results in preclinical trials. The aim of this study was to investigate the pharmacodynamic (PD) effects of three monoclonal microbial formulations of *L. lactis* spp. *cremoris* (EDP1066) on the immune response to keyhole limpet hemocyanin (KLH). Potential effects on the gut microbiota were also investigated.

**Methods:**

The trial was registered on Netherlands Trial Register (trial ID NL7519, https://trialsearch.who.int). Eighty-one healthy subjects (median 28, range 18–59 years) were randomized to 28 days of enteric-coated capsules at five doses (n = 13) (1.5 * 10^12^ total cells daily), freeze-dried powder at one dose (n = 12) (3.0 * 10^11^ total cells daily) or five doses (n = 12), minitablets at one dose (n = 12) or five doses (n = 12), or placebo (n = 20) prior to KLH immunization. Antibody responses and circulating regulatory T cells (Tregs) were measured after KLH immunization, and skin responses were evaluated after a KLH rechallenge by laser speckle contrast imaging and multispectral imaging. *Ex vivo* lymphocyte (phytohemagglutinin) and monocyte (lipopolysaccharide (LPS)) cytokine release assays were explored in the minitablet-treated groups only. The prevalence of *L. lactis* spp. *cremoris* in the gastrointestinal tract and the impact on the fecal microbiota were assessed by qPCR and 16S rRNA sequencing, respectively.

**Results:**

Repeated-measures analysis of covariances revealed no significant treatment effects on the antibody responses to KLH, number of Tregs, or KLH skin rechallenge outcomes. *Ex vivo* LPS-driven cytokine responses in whole blood were lower in the low dose minitablet group compared to placebo: tumor necrosis factor (estimated difference (ED) from placebo: −44.2%, 95% confidence interval (CI) −65.3% to −10.3%), interleukin (IL)-1β (ED −41.4%, 95% CI −63.5% to −5.8%), and IL-6 (ED −39.2%, 95% CI −56.8% to −14.5%). The fecal presence of *L. lactis* spp. *cremoris* increased during treatment by all EDP1066 formulations and normalized 5 days after the last dose. Microbiome α-diversity did not change by the treatments compared to placebo.

**Discussion:**

The EDP1066 formulations did not affect the immune response to KLH immunization in healthy individuals. However, exposure to *L. lactis* spp. *cremoris* in minitablet formulation impacted *ex vivo* whole blood LPS cytokine response. The clinical impact of these effects awaits further investigations.

**Netherlands Trial Register:**

trialsearch.who.int, trial ID NL7519.

## Introduction

Over the past decades, evidence has emerged for an interplay between the systemic immune system and the intestinal microbiome (1–3). The epithelium of the intestinal wall contains immune cells throughout, including in aggregated lymphoid nodules (Peyer’s patches), and the lamina propria and linked mesenteric lymph nodes (1, 4). Regional specialization of the gut immune network has been thoroughly studied in mice with differences found in antigenic composition, leukocyte populations, and gut-associated lymphoid tissue (GALT) (1). Although less evident, similar observations have been made in humans. The mucosa of the intestinal wall is also home to an abundance of microorganisms, and the composition and distribution of the microbial populations are dependent on the location within the gastrointestinal (GI) tract (1). Alterations in either the intestinal immune system or the gut microbiome can lead to various ailments such as celiac disease and inflammatory bowel disease (1, 5–7). Importantly, there is a growing body of evidence that hypothesizes that the effects of intestinal dysbiosis are not limited to local immunity and can also modify the immune response more distally as observed in systemic lupus erythematosus (8), rheumatoid arthritis (9), psoriasis (10), and more (11, 12). Altering the intestinal microbiota in these patient populations with intestinal dysbiosis, therefore, seems a plausible approach to evoke systemic immune modulation and consequently treat diseases associated with dysregulated immune responses. This hypothesis has been tested in more recent trials with orally administered probiotics (live microorganisms, when administered in adequate amounts, confer a health benefit on the host) (13, 14), prebiotics (non-digestible carbohydrates used as nutrients for probiotics), and/or synbiotics (blend of probiotics and prebiotics), which seem to have beneficial effects on dysregulated systemic immune responses (15–19), with some exceptions (15). Intake of certain probiotics has also been found to increase the responses to certain vaccinations (e.g., influenza) in humans depending on the choice, strain, dose, etc., of probiotics and vaccine type, dose, timing, and route (20). Interestingly, oral probiotics have also been demonstrated to be effective for the treatment of topical skin conditions, such as atopic dermatitis, acne, and rosacea (21), indicating induction of immune regulators. How oral administration of probiotic bacteria can modulate systemic immune responses and T cell-mediated inflammation in remote skin tissue is however unclear. Furthermore, studies using microbial strain mixtures suggest different immunomodulatory effects or even antagonism between species when compared with single-strain microbes, complicating the understanding of the underlying mechanisms (22–25).

One such single-strain microbial intervention is EDP1066, prepared from *Lactococcus lactis* spp. *cremoris* identified from powders used in dairy product manufacturing. Preclinical data of EDP1066 on both *in vitro* immune cell cultures and *in vivo* murine immune challenge and disease models show promising results; however, these data are not currently available in the public domain. In separate independent research, *L. lactis* spp. *cremoris* restored T-cell impairment in aged mice (26), and coadministration of *L. lactis* spp. *cremoris* with *Lactobacillus paracasei* spp. *paracasei* relieved atopic dermatitis symptoms, decreased serum IgE concentration, and rebalanced the population of Th1/Th2 cells in an atopic dermatitis mouse model (27).

Keyhole limpet hemocyanin (KLH) is a metalloprotein derived from the hemolymph of the marine mollusk, *Megathura crenulata*, which can be found in the Pacific coastal waters of California and Mexico. As the human body is unfamiliar with KLH, an *in vivo* immune response to this protein can be used to “mimic” an immune response to a pathogen or allergen in healthy volunteers (such as KLH-specific antibody formation and increased T-cell response after intradermal KLH rechallenge), providing essential information on proof-of-pharmacology during early-phase drug development (28–34). KLH was clinically introduced in 1967 to study the immunocompetence of humans (35) and since then is proven to be safe and widely used in clinical trials (28–31, 36–41).

The primary aim of the present study was to characterize the pharmacodynamic (PD) effects of EDP1066 on the systemic immune response to an intramuscular immunization with KLH and secondary to evaluate the effects on a subsequent KLH skin rechallenge. Because the exposure sites within the GI tract for ingested microbes may depend on the formulation and therefore be important for the immunomodulatory effect (1, 42), we also aimed at comparing different EDP1066 formulations (enteric-coated capsules, free freeze-dried powder, and minitablets) having different expected peak exposure sites. Furthermore, EDP1066 effects on numbers of circulating regulatory T cells (Tregs) were evaluated, and the *ex vivo* immunomodulatory activity of EDP1066 was explored by whole blood stimulation with the Toll-like receptor 4 ligand lipopolysaccharide (LPS) and phytohemagglutinin (PHA) for monocyte and lymphocyte stimulation, respectively. Finally, we aimed at assessing the impact of EDP1066 on the fecal microbiota, next to routine safety and tolerability assessments.

## Materials and methods

### Ethics

The independent Medical Ethics Committee “Medisch Ethische Toetsingscommissie van de Stichting Beoordeling Ethiek Biomedisch Onderzoek” (Assen, the Netherlands) approved the study prior to any clinical study activity. All subjects provided written informed consent before participation. The trial was registered on the Netherlands Trial Register, currently available for consultation at the International Clinical Trial Registry Platform (trial ID NL7519, https://trialsearch.who.int).

### Subjects

Healthy male and female participants were recruited *via* media advertisements and from the subjects’ database of the Centre for Human Drug Research, Leiden, the Netherlands. Enrolled participants were 18 to 60 years of age with a body mass index between 18 and 35 kg/m^2^ (2) and without previous exposure to KLH. Health status was verified by recording a detailed medical history, a complete physical examination, vital signs, a 12-lead electrocardiogram (ECG), and laboratory testing (including hepatic and renal panels, complete blood count, fecal calprotectin, virology, and urinalysis). Subjects were excluded in case of any disease associated with immune (e.g., active infection, auto-immune disease, primary or acquired immune deficiency, and clinically profound allergies) or GI system impairment (e.g., short bowel syndrome, diarrhea, inflammatory bowel disease, irritable bowel syndrome, and celiac disease) or use of prescription medication within 4 weeks prior to the first dose. Other exclusion criteria were antibiotic treatment within 42 days prior to initial dosing and during the course of the study and the use of probiotic capsules within 14 days of screening and during the course of the study.

### Dose selection and regimen

All EDP1066 and placebo formulations were manufactured and provided by Evelo Biosciences Inc. (Cambridge, MA, USA). The doses tested were based on the results of a separate first-in-man study (43). The highest dose tested contained 1.5 * 10^12^ total cells per dose, approximately five times the predicted therapeutic dose level, calculated from allometric scaling of the preclinically efficacious dose level based on conversion between mouse and human gut surface area. This dose was well tolerated in humans. Three different formulations of the investigational drug were investigated: enteric-coated capsules containing EDP1066 freeze-dried powder, EDP1066 as free freeze-dried powder, and non-coated capsules containing enteric-coated EDP1066 minitablets. For each EDP1066 formulation, matching placebo formulations were used in order to preserve the blinding. The three placebo formulations contained similar excipients as their active treatment counterparts, without the EDP1066 microbes. The excipients present in the three EDP1066/placebo formulations (e.g., microcrystalline cellulose, magnesium stearate, mannitol, citric acid, and sodium hydroxide) are widely used in drug product manufacturing, and none of the excipients were expected to elicit immune system modulation.

### Study design and treatments

This was a phase 1, randomized, placebo-controlled, double-blind, multiple-dose study in 80 healthy volunteers performed at the Centre for Human Drug Research (CHDR), Leiden, the Netherlands, based on the principles of the Declaration of Helsinki. An overview of the study design is shown in **Table 1**. Participants were randomized to one out of the five groups of EDP1066 or placebo (12:4 per group) in a consecutive order starting with the lowest number. The randomization code was computer-generated by a study-independent statistician and was only made available for data analysis after study completion. One group received EDP1066 freeze-dried powder in enteric-coated capsules, supplied as 1.5 * 10^11^ total cells per capsule, administered orally at a dose of 10 capsules daily (5× Capsules). Two other groups received EDP1066 as free freeze-dried powder with an achlorhydria regimen administered orally at a dose of 3.0 * 10^11^ (1× Powder) and 1.5 * 10^12^ (5× Powder) total cells daily. The achlorhydria regimen consisted of omeprazole 40 mg and aluminum hydroxide/magnesium hydroxide 200/400 mg administration 3 h prior to each EDP1066 dose. Both drugs increase the gastric pH (44–46) and were expected to improve the transition of EDP1066 through the stomach and into the duodenum. Omeprazole and aluminum hydroxide/magnesium hydroxide are not known to induce immune system modulation. Another two groups received non-coated capsules containing enteric-coated EDP1066 minitablets, supplied as 1.5 * 10^11^ total cells per capsule, administered orally at a dose of 2 (1× Minitablets) and 10 (5× Minitablets) capsules daily. Participants were dosed once daily for 28 consecutive days. Compliance was confirmed by the supervised administration of the study treatment during the in-clinic period. Administration at home was recorded by an electronic diary. Intramuscular KLH immunization was performed in the left deltoid muscle after the completion of the third administration of EDP1066/placebo. KLH immunization was administered as 0.1 mg of Immucothel^®^ adsorbed in 0.9 mg of aluminum hydroxide (Alhydrogel^®^) into 0.5 ml of NaCl 0.9% as previously described (47). All subjects were administered KLH (0.001 mg of Immucothel^®^) and saline in 0.1 ml of NaCl 0.9% intradermally in the left and right ventral forearms, respectively, 23 days after KLH immunization. The skin challenge response was quantified prior to and 2 days after intradermal KLH administration. These are similar intervals between assessments as in our previous studies, which also detail the methodology (29, 32, 36, 38, 41, 47, 48). To account for ambient and environmental factors, the responses observed at the intradermal KLH administration site were corrected against the intradermal saline administration site on the contralateral forearm. A follow-up visit 5 days after the last EDP1066/placebo dose and a study discharge visit 12 days after the last EDP1066/placebo dose were included in order to assess EDP1066 stool persistence and prevalence and EDP1066 effects on the gut microbiome.

**Table 1:** Study timeline.

		Treatment									FU
	Timepoint	D -1	D 1	D 3	D 5	D 10	D 17	D 26	D 28	D 33	D 40
Activity											
EDP1066 / placebo administration			←-----------------Once daily----------------→								
KLH immunization				X							
Anti-KLH IgM and IgG				X		X	X	X			X
Tregs + *ex vivo* stimulation assays			X	X	X	X		X			X
Intradermal KLH administration								X			
Intradermal KLH readout (LSCI, MI)									X		
Fecal EDP1066 concentration		X						X		X	
Fecal microbiome		X								X	X
Admission		←---------------------→									

X indicates performed activity.

D, day; KLH, keyhole limpet hemocyanin; Tregs, regulatory T cells; LSCI, laser speckle contrast imaging; MI, multispectral imaging; FU, follow up.

### Humoral immunity to keyhole limpet hemocyanin

The humoral response to KLH immunization was measured by anti-KLH IgM and IgG serum titers. Serum samples for the analysis of anti-KLH IgM and IgG were obtained in non-additive tubes by venipuncture at the time points indicated in **Table 1**. Samples were centrifuged at 2,000 *g* for 10 min with a temperature of 2°C–8°C, and the serum was aliquoted. The aliquots were stored at a temperature of −40°C until shipment and analysis. Samples were assessed by quantitative enzyme-linked immunosorbent assay (ELISA) for anti-KLH IgM and IgG as previously described (ELISA developed in-house by Ardena Bioanalytical Laboratory (Assen, the Netherlands)) (47). For the analysis of human antibodies raised against KLH, no reference material was available for the preparation of calibration standards and quality checks. Quantitative measurement of human anti-KLH IgG and IgM (in µg/ml) using a standard curve was not an option. Therefore, the mean optical density of baseline samples was set to 1.00, and relative ratios were calculated for all subsequent samples.

### Cutaneous blood perfusion and erythema

Cutaneous blood perfusion quantification was performed with laser speckle contrast imaging (LSCI) (PeriCam PSI System, Perimed AB, Järfälla, Sweden), and erythema quantification was performed with multispectral imaging (Antera 3D^®^, Miravex, Dublin, Ireland) as previously described (47). Circular regions of interest at the intradermal injection sites were defined. Cutaneous blood perfusion (indicated as basal flow) was quantitatively assessed and expressed in arbitrary units (AUs). The homogeneity of cutaneous blood perfusion in the region of interest (indicated as flare), expressed as values that are +1 standard deviation (SD) from the mean basal flow within the region, was also quantitatively assessed and expressed in AUs. Erythema was quantified using the average redness and CIELab a* Antera 3D^®^ software modalities expressed as AUs. The average redness modality displays the distribution of redness using an internal software algorithm, and the CIELab a* value, which is part of the CIELab color space, expresses color as a numerical value on a green–red color scale.

### Circulating regulatory T cells and *ex vivo* stimulation assays

The percentage of circulating Tregs was evaluated by flow cytometry. Venous blood was collected in sodium heparin tubes by venipuncture at the time points indicated in **Table 1**. Red blood cell (RBC) lysis was performed on heparinized whole blood using RBC lysis buffer (Thermo Fisher, Waltham, MA, USA). Leukocytes were stained with fluorochrome-labeled antibodies CD4-VioBlue, CD25-APC, and CD127-PE; propidium iodide was used as viability dye (all Miltenyi Biotec, Bergisch-Gladbach, Germany). Samples were analyzed on a MACSQuant 16 analyzer (Miltenyi Biotec) using FlowLogic software (Inivai, Mentone, VIC, Australia). Tregs were defined as CD4^+^CD25^+^CD127^−^; see **Figure S1** for the gating strategy. *Ex vivo* lymphocyte and monocyte cytokine release assays were incorporated later in the study to examine NF-κB-driven responses and only performed in the minitablet-treated groups in which the most optimal immunomodulatory results were expected based on preclinical data. Sodium heparinized whole blood was incubated with 10 µg/ml of PHA (Sigma-Aldrich, Deisenhofen, Germany) or 2 ng/ml of LPS (strain O111:B4 from *Escherichia coli*, Sigma-Aldrich) for 24 h at 37°C, 5% CO_2_. After 24 h, the supernatant was collected, and cytokines were measured using qualified ELISA-based assays by Ardena Bioanalytical Laboratory. Interferon gamma (IFN-γ) and IL-2 were measured in the PHA-stimulated samples; tumor necrosis factor (TNF), IFN-γ, IL-1β, IL-6, and IL-8 were measured in the LPS-stimulated samples.

### EDP1066 stool persistence and gut microbiome

Fecal concentrations of EDP1066 for stool persistence and prevalence and the gut microbiome were measured by Diversigen Inc. (Houston, TX, USA) using validated bioanalytical assay methods. In short, fecal microbial DNA was extracted based on the Zymo Research (Irvine, CA, USA) fecal DNA extraction methodology. EDP1066-specific primers and probes had been developed to enable the detection of the *L. lactis* spp. *cremoris* strain. The fecal samples were analyzed using a qPCR with a lower limit of quantification of 5.0 copies/5 ng DNA. For gut microbiome analyses, extracted DNA was prepared for Illumina sequencing *via* PCR amplification of the variable region 4 of the bacterial 16S rRNA gene. After PCR purification using AMPure XP beads (Beckman Coulter Life Sciences, Indianapolis, IN, USA), sample-specific barcodes using Illumina Nextera XT Index kit (Illumina Inc., San Diego, CA, USA) were appended to the PCR products during a second PCR. The PCR products were purified for a second time, and lastly, the PCR products were equimolarly pooled and sequenced on the Illumina MiSeq platform using the MiSeq v3 sequencing kit.

### Safety and tolerability

Safety and tolerability were monitored by physical examination, assessment of vital signs, laboratory parameters (i.e., full blood count, biochemistry, serology, immunophenotyping, circulating cytokines, fecal calprotectin, and urinalysis), and ECG data from 12-lead ECGs at regular intervals. Subjects were monitored continuously for adverse events (AEs). Participants were also asked to daily complete the Bristol Stool Scale (BSS) and questions regarding defecation patterns using an electronic diary app in order to obtain insight into the participants’ stool patterns at the time of fecal sample collection.

### Statistics

The sample size was based on previously performed power calculations on KLH challenge endpoints (47). In order to detect a 75% inhibition of the KLH-specific antibody response, cutaneous blood perfusion response (LSCI), and erythema response (multispectral imaging), a sample size of 12 per group was required using a parallel study design, with an α of 0.05 and a power of 80%. It was deemed appropriate to pool the placebo-treated participants for analyses in order to increase the statistical power. Demographic and baseline variables were summarized by treatment. PD endpoints measured at multiple time points after baseline were analyzed with a mixed-effects repeated-measures model with fixed factors treatment, time and treatment by time, random factor subject, and the baseline value as covariates. Endpoints with one post-dose measurement were analyzed with a linear model with treatment as a fixed factor. Anti-KLH antibody parameters were analyzed without baseline as a covariate. Skin rechallenge endpoints were analyzed with an analysis of covariance with treatment as a fixed factor and the baseline and the change from baseline (CFB) of the saline-injected control added as covariates. Anti-KLH IgM and IgG titers and *ex vivo* monocyte cytokine release assays required log transformation. The general treatment effect and specific contrasts were reported as the estimated difference (ED) with a 95% confidence interval (CI) and graphically as ED with 95% CI, as least squares mean (LSM) with 95% CI, or as mean with SD. Fecal EDP1066 concentration was reported graphically as median with range. Fecal microbiome endpoints were analyzed using Python (Python Software Foundation, Wilmington, DE, USA) by Diversigen Inc. Read count and relative abundance tables were calculated at the genus level and retrieved using custom Python scripts and the One Codex Python library, an in-house curated database of bacterial marker genes including 16S ribosomal RNA. The relative abundances of all microorganisms at the genus level were calculated to present the occurrence of the *Lactococcus* genus relative to all microbial DNA in the samples. Diversity trend analysis was performed using the Shannon diversity index. The Shannon diversity index was calculated for all samples using the One Codex Python library. Results were aggregated and plotted using custom Python scripts. To determine whether some genera were more or less abundant in placebo *vs.* EDP1066 treated individuals, read count tables were fed to ANCOM, a statistical framework for the analysis of microbiomes. Fecal microbiome diversity was reported graphically as median with an interquartile range.

## Results

### Baseline characteristics

**Figure 1:**
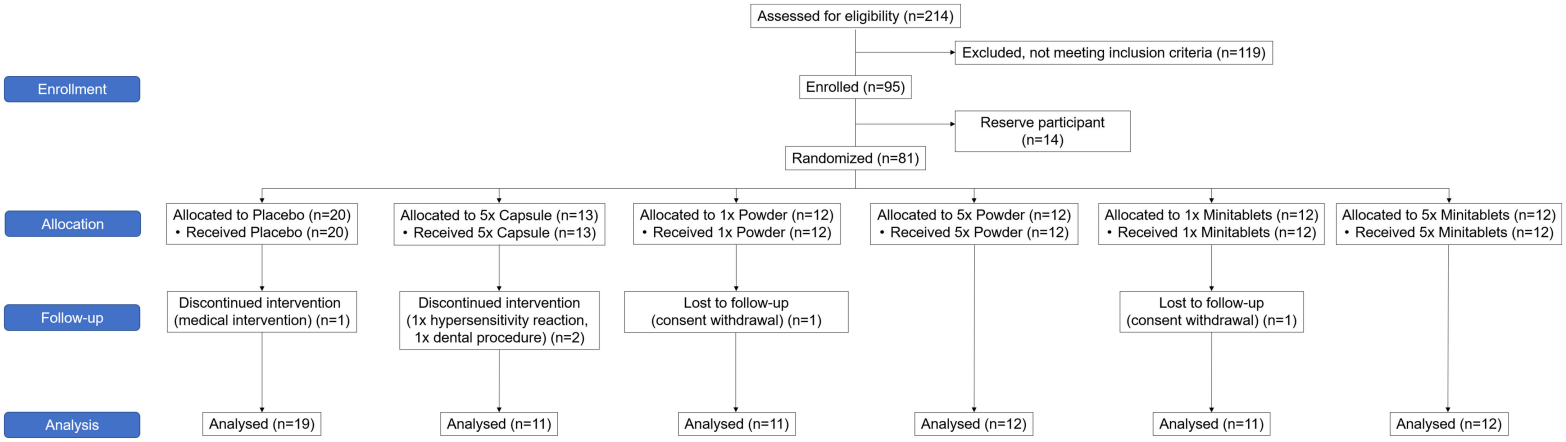
CONSORT 2010 flow diagram of the progress through the phases of the trial.

The study was conducted between February 2019 and January 2020. Ninety-five subjects were enrolled in the study of which 81 were treated (**Figure 1**). A total of 76 subjects completed the treatment and the follow-up period. Five subjects did not complete the study. One subject was withdrawn due to a possible hypersensitivity reaction to EDP1066. Due to very limited EDP1066 exposure (two doses) and collected data, it was decided to replace this subject. The withdrawal in the other four was unrelated to the study drug or procedures (emergency dental procedure (one), tetanus vaccination and antibiotics treatment (one), and consent withdrawal (two)). The baseline characteristics of all treatment groups are presented in **Table 2**. Treatment compliance was 99.4% in subjects who completed the treatment and follow-up period (range number of days EDP1066 intake 26–28 days). Nine subjects missed one dosing day, and two subjects missed two dosing days.

**Table 2:** Baseline characteristics.

EDP1066 FORMULATION	Enteric-coated capsules	Free powder		Minitablets		Placebo
DAILY DOSE	1.5*10^12^ (5x) total cells	3.0*10^11^ (1x) total cells	1.5*10^12^ (5x) total cells	3.0*10^11^ (1x) total cells	1.5*10^12^ (5x) total cells	
	n=13	n=12	n=12	n=12	n=12	n=20
**DEMOGRPAHICS**						
Age (years)	30 (18-59)	26 (19-58)	29 (20-59)	25 (18-56)	51 (22-56)	26 (19-59)
BMI (kg/m^2^)	24.5 (3.8)	23.7 (2.7)	26.0 (4.4)	21.9 (3.0)	22.0 (2.6)	24.3 (3.7)
Male gender (n)	9 (69.2%)	6 (50.0%)	8 (66.7%)	6 (50.0%)	6 (50.0%)	10 (50.0%)
**VITAL SIGNS**						
Systolic blood pressure (mmHg)	117 (13)	111 (9)	118 (11)	109 (13)	110 (10)	110 (8)
Diastolic blood pressure (mmHg)	68 (10)	65 (9)	69 (9)	64 (10)	66 (8)	64 (6)
Heart rate (bpm)	60 (12)	56 (9)	59 (8)	62 (8)	61 (5)	56 (7)
Temperature (°C)	36.4 (0.3)	36.5 (0.3)	36.5 (0.3)	36.6 (0.3)	36.4 (0.5)	36.5 (0.5)
**LABORATORY TESTS**						
Leucocytes (*10^9^/L)	7.47 (2.00)	7.00 (1.50)	7.55 (1.79)	7.27 (2.42)	7.35 (1.34)	6.71 (1.53)
Lymphocytes (*10^9^/L)	2.25 (0.49)	2.22 (0.55)	2.46 (0.50)	2.38 (0.81)	2.55 (0.73)	2.35 (0.77)
Thrombocytes (*10^9^/L)	276.7 (61.3)	254.8 (49.3)	251.9 (35.4)	230.8 (48.7)	253.5 (64.0)	259.3 (46.4)
ALT (IU/L)	21.4 (6.7)	24.4 (8.9)	24.6 (10.4)	20.6 (13.0)	25.6 (11.3)	19.8 (7.7)
AST (IU/L)	20.3 (3.3)	22.4 (7.4)	23.9 (9.2)	20.1 (4.6)	25.7 (5.2)	20.4 (5.9)
CRP (mg/L)	1.05 (1.34)	1.56 (2.36)	1.41 (1.43)	1.58 (2.05)	0.53 (0.50)	1.61 (1.70)
Fecal calprotectin (µg/g)	34.0 (36.2)	11.0 (12.9)	9.0 (11.9)	17.4 (14.6)	16.8 (11.9)	18.0 (15.3)

Parameters are shown as mean (standard deviation), age as median (range), and male gender as count (percentage).

ALT, alanine transaminase; AST, aspartate transaminase; CRP, C-reactive protein; BMI, body mass index.

### Humoral immunity to keyhole limpet hemocyanin and cutaneous blood perfusion and erythema

**Figure 2:**
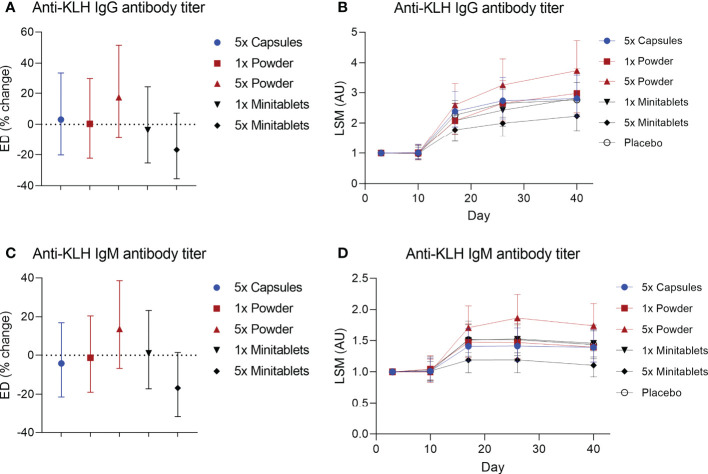
Anti-keyhole limpet hemocyanin **(A, B)** IgG and **(C, D)** IgM antibody titers by EDP1066 treatment group. Data are shown as estimated difference with 95% confidence interval expressed as percentage of placebo in panels **(A, C)** and as least square means with 95% confidence interval in panels **(B, D)** The estimated difference was calculated with a mixed-effects repeated-measures model with fixed factors treatment, time and treatment by time, and random factor subject as covariate. KLH, keyhole limpet hemocyanin; ED, estimated difference; LSM, least square means.

**Figure 3:**
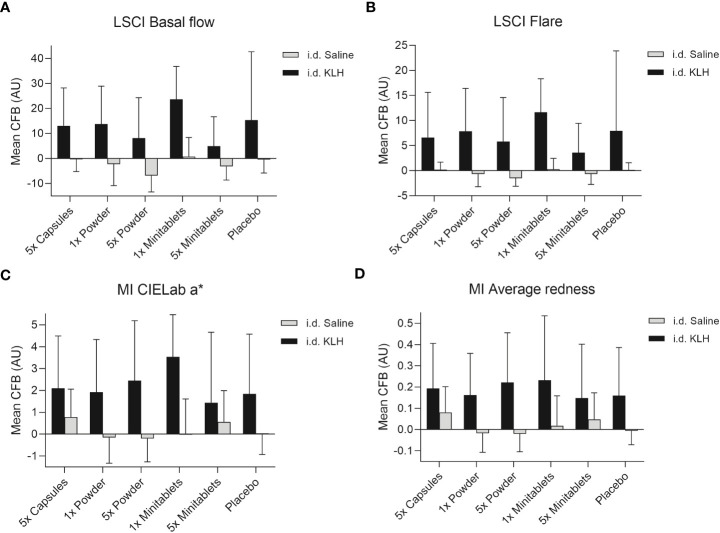
Cutaneous blood perfusion by **(A)** LSCI basal flow and **(B)** LSCI flare, erythema by **(C)** CIELab a* (multispectral imaging), and **(D)** average redness (multispectral imaging) after intradermal KLH and saline administration by three EDP1066 formulations of *Lactococcus lactis* spp. *cremoris*: i) enteric-coated capsules, ii) freeze-dried powder (dose 1× and 5×), and iii) minitablets (dose 1× and 5×). The average redness modality displays the distribution of redness using an internal software algorithm and the CIELab a* value, which is part of the CIELab color space, expresses color as a numerical value on a green–red color scale. Data are shown as mean change from baseline (CFB) with standard deviation. LSCI, laser speckle contrast imaging; MI, multispectral imaging; KLH, keyhole limpet hemocyanin; CFB, change from baseline; AU, arbitrary unit; i.d., intradermal.

No statistically significant treatment or formulation effects were observed on the humoral KLH challenge outcomes. Observations closest to the desired treatment effect were lower anti-KLH IgG (**Figure 2**, ED −16.8%, 95% CI −35.5% to 7.3%, p = 0.15) and IgM (**Figure 2**, ED −16.8%, 95% CI −31.8% to 1.4%, p = 0.07) levels in the 5× Minitablets group compared to placebo, not reaching a level of statistical significance. No statistically significant treatment or formulation effects were observed on the KLH skin rechallenge outcomes (**Figure 3**).

### Circulating regulatory T cells and *ex vivo* stimulation assays

**Figure 4:**
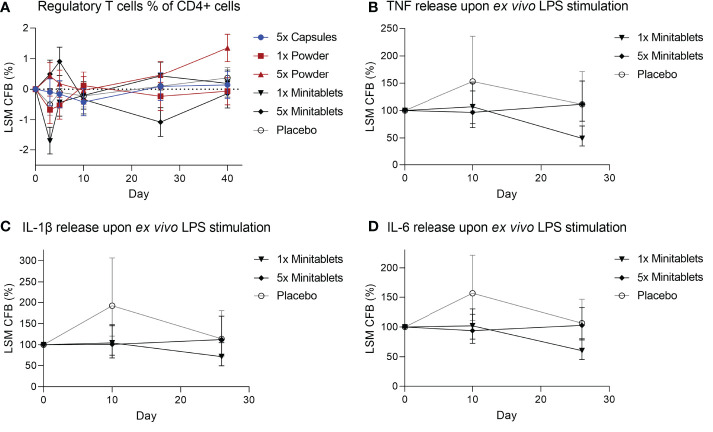
**(A)** Circulating regulatory T cells as percentage of CD4^+^ T cells from heparinized blood. Monocyte cytokine release assay of **(B)** tumor necrosis factor, **(C)** interleukin-1β, and **(D)** interleukin-6 release from whole blood cultures after *ex vivo* lipopolysaccharide stimulation. X-axis represents number of days after initial EDP1066 dose. Data are shown as least squares mean change from baseline **(**CFB) with 95% confidence interval. LSM, least squares mean; CFB, change from baseline; LPS, lipopolysaccharide; TNF, tumor necrosis factor; IL = interleukin.

There were no consistent EDP1066-dependent changes in the percentage of circulating Tregs over all groups, though Tregs were significantly increased in subjects treated with 5× Powder (**Figure 4**, ED 0.55%, 95% CI 0.14%–0.96%, p < 0.01) compared to placebo. EDP1066 slightly impacted LPS-driven cytokine release in whole blood cultures. Overall, all cytokines (IFN-γ, IL-1β, IL-6, IL-8, and TNF) in supernatants from LPS-stimulated whole blood cultures were lower in the 1× and 5× Minitablets groups compared to placebo. Furthermore, a statistically significant decreased TNF (**Figure 4**, ED −44.2%, 95% CI −65.3% to −10.3%, p < 0.05), IL-1β (**Figure 4**, ED −41.4%, 95% CI −63.5% to −5.8%, p < 0.05), and IL-6 (**Figure 4**, ED −39.2%, 95% CI −56.8% to −14.5%, p < 0.01) release were observed in the 1× Minitablet group compared to placebo. There were no consistent findings in supernatants of PHA-stimulated whole blood samples over the biomarkers and groups.

### EDP1066 stool persistence and gut microbiome

**Figure 5:**
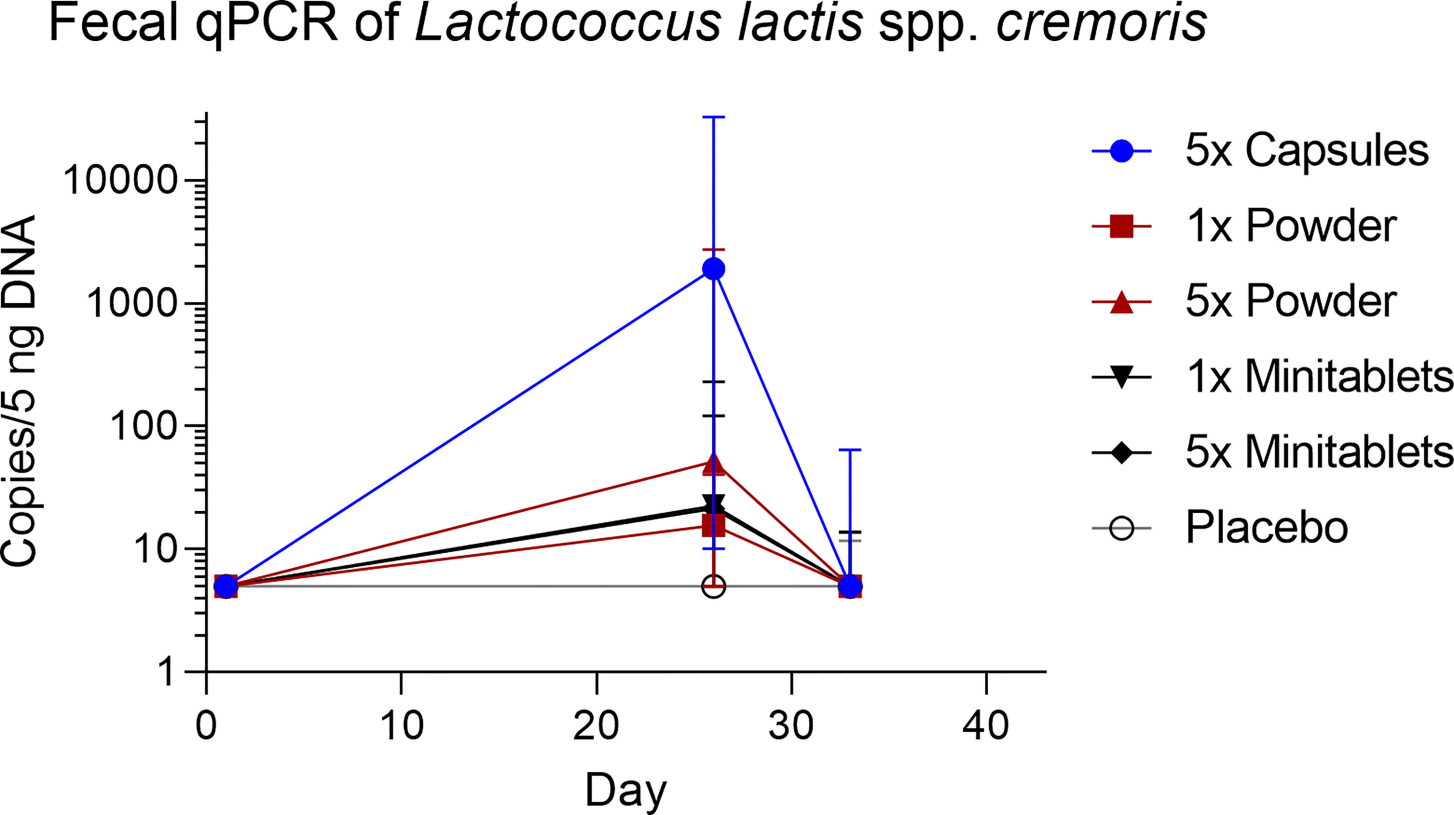
Fecal concentration of *Lactococcus lactis* spp. *cremoris* measured by quantitative polymerase chain reaction. Data are shown as median with range. qPCR, quantitative polymerase chain reaction.

**Figure 6:**
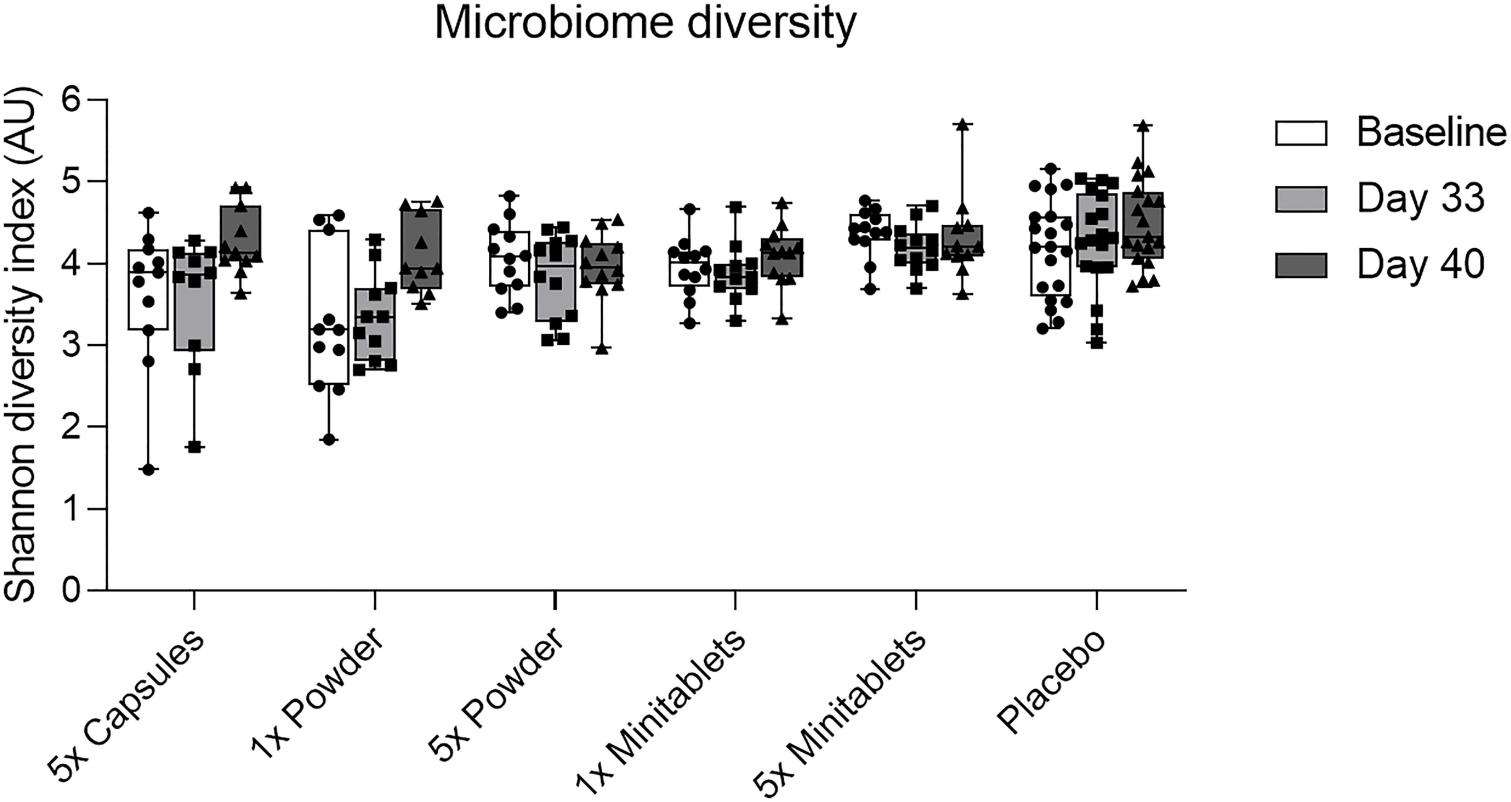
Fecal microbiome diversity calculated using the Shannon diversity index by treatment group per time point. Data are shown as median with interquartile range. AU, arbitrary unit.

*L. lactis* spp. *cremoris* was detected in all actively treated groups in 64% to 73% of subjects on study day 26. Levels returned toward baseline 5 days after the last EDP1066 dose (**Figure 5**). Dosing by 5× Capsules formulation resulted in the detection of fecal *L. lactis* spp. *cremoris* in all subjects on study day 26 (**Figure 5**). *Lactococcus* genera were represented only in trace amounts in all samples (**Figure S2**). The maximum number of *Lactococcus* reads detected in any of the subjects was approximately 500, which corresponds to 0.6% of the total classified 16S reads. These results suggest that EDP1066 did not colonize the gut of any of the participants. Microbiome diversity (Shannon diversity index) was comparable among time points and treatment groups, albeit some changes could be observed on individual levels for a subset of the participants (data not shown). Overall microbiome diversity seemed to be slightly lower in EDP1066-treated samples; however, many of these differences probably occured due to the small sample size when calculations were performed for individual groups. When Shannon diversity indices were aggregated across all the groups, the mean Shannon diversity was very stable between time points and treatment groups (**Figure 6**). The 10 most abundant genera were very stable between EDP1066- and placebo-treated subjects (**Figure S3**). There was some variation in relative abundance, but no large or consistent shifts were seen across all groups. Variation was most likely due to individual differences in microbiome composition between subjects and not dependent on treatment.

### Safety and tolerability

Overall, no major safety concerns were observed during the study. No serious adverse events occurred. Most AEs were related to the GI tract (93 AEs in 46 subjects) with no distinction between EDP1066 and placebo treatment (Table S1). One subject was withdrawn from further treatment after the second EDP1066 dose due to a possible hypersensitivity reaction to EDP1066 consisting of a mild burning sensation and itch of the throat lasting approximately 6 h. No abnormalities were found upon physical examination and additional vital sign measurements. Due to the mild and limited nature of the AEs, no further diagnostics were conducted. No earlier hypersensitivity AEs after EDP1066 administration had been reported. The subject also did not report any allergies to cheese or other dairy products. Allergic reactions to excipients used in the 5× Capsule formulation (microcrystalline cellulose, magnesium stearate, and colloidal silicon dioxide) have been reported before; however, these are very rare (49–51). Placebo-treated subjects had slightly fewer AEs (75%) compared to EDP1066-treated subjects (83.3% to 91.7%). No clinically significant changes were observed in laboratory parameters, vital signs, ECG recordings, and the BSS and feces questionnaire.

## Discussion

In this study, we showed that daily EDP1066 treatment in encapsulated, powdered, and minitablet formulations and daily doses up to 1.5 * 10^12^ total cells, five times the expected therapeutic dose, did not result in consistent significant effects on KLH challenge responses and LPS- and PHA-driven cytokine release in whole blood cultures. We demonstrated that *L. lactis* spp. *cremoris* was detected in the fecal samples and increased during the 28-day treatment period for all EDP1066 formulations tested. However, the fecal levels returned to baseline levels 12 days after the end of treatment, indicating no prolonged persistence. Overall, EDP1066 was considered safe and well-tolerated. To the best of our knowledge, the current trial is the first to investigate the effects of orally administered *L. lactis* spp. *cremoris* in high doses on systemic immune responses and the gut microbiome.

EDP1066 did not show a consistent immunomodulatory effect on KLH-driven responses in the present study. Though no statistical significance was reached, decreased anti-KLH antibody titers and cutaneous blood perfusion and erythema were observed in the 5× Minitablets group compared to placebo. Although circulating Tregs as a percentage of CD4^+ ^T cells were significantly increased in subjects treated with 5× Powder compared to placebo, it should be noted that these percentages remained within the general range of Tregs in the CD4 population as reported in the literature (5%–10%) (52, 53). The PD results observed in this study are in contrast with preclinical data where EDP1066 induced IL-10 production in *in vitro* human dendritic cell (DC) cultures, without significant induction of pro-inflammatory cytokines (unpublished data), and EDP1066 significantly reduced KLH- and ovalbumin-induced ear inflammation in mice and improved intestinal pathology and weight loss in an acute dextran sulfate sodium-induced colitis mouse model (unpublished data). Also in contrast to our results, other preclinical trials reported that *L. lactis* spp. *cremoris* restored T-cell impairment in aged mice (26) and that coadministration of *L. lactis* spp. *cremoris* with *L. paracasei* spp. *paracasei* showed promising results in an atopic dermatitis mouse model (27). Probiotics in general have been shown to be effective in (the prevention of) multiple diseases (20, 25). Multiple studies have reported enhanced responses to influenza vaccination after the intake of probiotics (54–58). Another study showed an enhanced response to hepatitis A vaccination after probiotic intake (59). Single strains of both *Lactobacillus rhamnosus GG* and *Lactobacillus helveticus R52* have been shown to reduce the risk of developing antibiotic-associated diarrhea (25). *L. rhamnosus GG* single-strain treatment was also effective in the prevention of necrotizing enterocolitis (25). Furthermore, *Bifidobacterium animalis* spp. *lactis Bb12* prevented upper respiratory tract infections, indicating distally evoked immune system effects (25).

EDP1066 treatment suppressed KLH-driven increases in LPS-driven cytokine release *ex vivo* in both the 1× and the 5× Minitablets groups, reaching statistical significance for IL-1β, IL-6, and TNF-α in the 1× Minitablets group, which may indicate innate immune system inhibition (60). The observed increase in LPS-driven cytokine release by monocytes in placebo-treated subjects may be attributed to KLH immunization, priming the innate immune response for subsequent stimulation. Although KLH is primarily recognized as an agent that induces cell-mediated responses, there is evidence that KLH immunization and rechallenge most likely cause a mixed reaction of innate, late-phase skin reaction and delayed-type hypersensitivity (61). Furthermore, similar to LPS, KLH induces innate immunity *via* the activation of NF-κB (60).

In the present study, we evaluated the immunomodulatory activity of EDP1066 as powder formulation (free and in enteric-coated capsules) and as minitablets in non-coated capsules. The minitablets in non-coated capsule formulation were expected to achieve the highest concentration of relatively intact EDP1066 bacteria in the duodenum. Non-coated capsules were used to ease the intake of relatively large numbers of enteric-coated minitablets and to preserve the blinding. Based on *in vitro* experiments, the minitablet formulation was predicted to release in the proximal small intestine (unpublished data). Duodenal EDP1066 exposure was hypothesized to be important for the immunomodulatory effect, as immune cell subsets are found at the highest concentrations in the duodenum and jejunum, particularly the CD103^+^CD11b^+^ DCs, which are thought to play distinct roles in intestinal immune homeostasis (1). The small intestine is the most likely point where luminal contents can access GALT and have a pronounced immune-regulatory effect (1). However, we did not observe any differences between the formulations on the humoral KLH challenge and subsequent skin KLH rechallenge, circulating Tregs, gut microbiome, and safety and tolerability outcomes. We did observe increased fecal detection of EDP1066 in the capsule formulation compared to powder and minitablet formulations. This can possibly be explained by the fact that the enteric-coated capsules dissolve lower in the GI tract leading to postponed EDP1066 release and higher EDP1066 exposure toward the end of the GI tract.

The current human trial did not confirm previous findings from preclinical trials that oral administration of EDP1066 had immunomodulatory effects as measured on antibody response to KLH immunization or skin immune responses to KLH re-challenge. There are several potential explanations for the suboptimal translation of EDP1066 activity between mice and humans. Firstly, it was impossible to do conventional allometric scaling between mice and humans. Other than for most medicinal products, the exposure to EDP1066 was considered to remain restricted to the GI tract. This was hypothesized to be sufficient, since the mechanism of action of EDP1066 only requires local interaction with cells of the GI mucosa, driving subsequent systemic effects. Under these conditions, assumptions of traditional allometric scaling may not hold true. For this reason, relative GI mucosal surface area and stool mass are key parameters for allometric scaling. The relative GI mucosal surface area has been estimated as a function of body mass to the ¾ power (62). As the dose selection rationale was mainly hypothetical, the actual EDP1066 doses administered might have been too low to exert significant PD effects. For practical reasons, the administration of higher EDP1066 doses was not explored since this would require a daily intake of >10 capsules. Secondly, differences in diet and also differences in microbial composition are likely to introduce highly variable individual responses to microbial exposure. As *L. lactis* spp. *cremoris* is used by the dairy industry, it is likely that participants have developed at least some intestinal tolerance to this microbe (63), possibly explaining the differences observed between the current trial and preclinical data. Furthermore, apart from the well-known immunological differences between rodents and humans, EDP1066 activity is dependent on relatively unknown physiological systems or principles such as the GI microbiome and the interplay between the local and systemic immune systems. The exact molecular target and target location for EDP1066 are unknown, as are the exact EDP1066 components required for biological effect, further complicating inter-species translation. As intestinal dysbiosis can cause altered local as well as systemic immune system changes, we hypothesized that live EDP1066 would be required to interact with the gut microbiome and local immune system. However, based on the results observed in this study, we cannot exclude the possibility that dead EDP1066 could potentially also provoke immune system responses. Finally, the *in vitro* prediction of the release criteria of the enteric-coated capsules and minitablets may underestimate the time to release, suggesting that the true release of EDP1066 was in the distal small intestine or colon rather than in the proximal small intestine.

In conclusion, oral EDP1066 treatment for healthy volunteers did not consistently result in significant immune modulation. Future clinical studies should build onto the insights obtained in this study and further investigate formulation versus local release and dose–effect relationships, which will ultimately be beneficial not only for EDP1066 but also for the field of therapeutic human commensals in general.

## Data availability statement

The datasets for this article are not publicly available due to concerns regarding participant/patient anonymity. Requests to access the datasets should be directed to the corresponding author.

## Ethics statement

The studies involving human participants were reviewed and approved by Medisch Ethische Toetsingscommissie van de Stichting Beoordeling Ethiek Biomedisch Onderzoek. The patients/participants provided their written informed consent to participate in this study.

## Author contributions

All authors wrote the manuscript. AI, DM, MM, PG, and MS designed the research. MM, PG, and MS performed the research. HG and EK analyzed the data. All authors approved the final version of the manuscript.
