# Supplementary material for: Impact of oral administration of single strain Lactococcus lactis spp. cremoris on immune responses to keyhole limpet hemocyanin immunization and gut microbiota: A randomized placebo-controlled trial in healthy volunteers
Source: Front Immunol. 2022 Dec 7;13:1009304. doi: 10.3389/fimmu.2022.1009304 (PMC9793106; doi:10.3389/fimmu.2022.1009304)
Supplement: Supplementary file 1 [file Image_1.pdf]

Supplementary Material

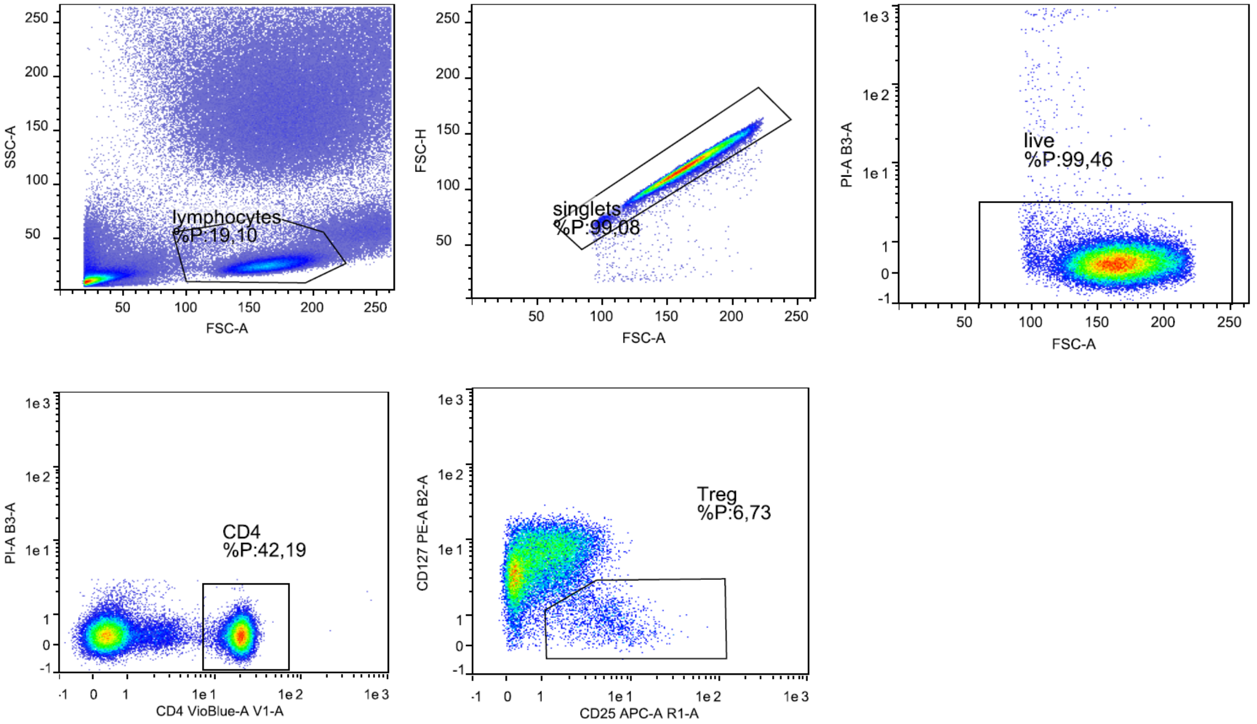

Figure S1. Gating strategy of circulating regulatory T cells. Lymphocytes were gated in the FSC/SSC plot, followed by gating of singlets and subsequently live cells using propidium iodide as viability dye. $CD4^+$ cells were gated thereafter and regulatory T cells were assessed as $CD25^+$ and CD1.
