# Supplementary material for: Impact of oral administration of single strain Lactococcus lactis spp. cremoris on immune responses to keyhole limpet hemocyanin immunization and gut microbiota: A randomized placebo-controlled trial in healthy volunteers
Source: Front Immunol. 2022 Dec 7;13:1009304. doi: 10.3389/fimmu.2022.1009304 (PMC9793106; doi:10.3389/fimmu.2022.1009304)
Supplement: Supplementary file 3 [file Image_3.pdf]

Supplementary Material

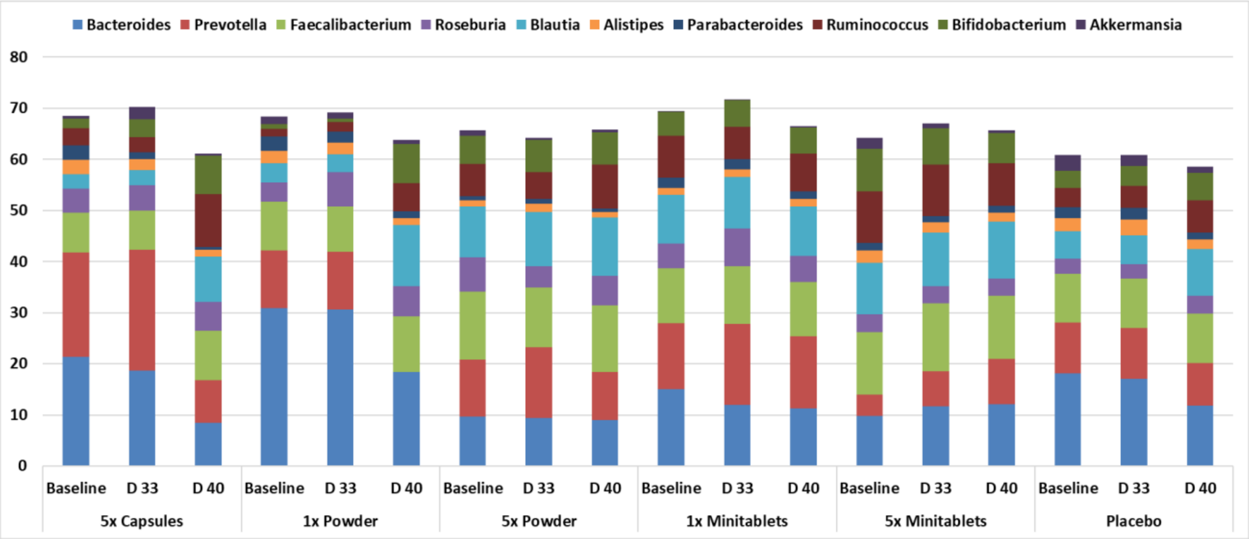

Figure S3. Distribution of the ten most abundant microbial genera expressed as percentage composition of total microbiome summarized per treatment and displayed per time point per group. D 33 = Day 33, D 40 = Day 40.
