# Supplementary material for: Impact of oral administration of single strain Lactococcus lactis spp. cremoris on immune responses to keyhole limpet hemocyanin immunization and gut microbiota: A randomized placebo-controlled trial in healthy volunteers
Source: Front Immunol. 2022 Dec 7;13:1009304. doi: 10.3389/fimmu.2022.1009304 (PMC9793106; doi:10.3389/fimmu.2022.1009304)
Supplement: Supplementary file 4 [file Table_1.pdf]

Supplementary Material

Table S1: Summary of number of subjects with treatment emergent adverse events by treatment, System Organ Class and severity. Mil = Mild, Mod = Moderate, n = number of subjects, % = percentage of subjects.

System Organ Class		5x Capsules<br>Events (n, %)	1x Powder<br>Events (n, %)	5x Powder<br>Events (n, %)	1x Minitablets<br>Events (n, %)	5x Minitablets<br>Events (n, %)	Placebo<br>Events (n, %)
Any events		39 (11, 84,6%)	33 (11, 91.7%)	39 (11, 91.7%)	21 (10, 83.3%)	38 (11, 91.7%)	57 (15, 75.0%)
	Severity (n)	*Mil (11)*<br>*Mod (5)*	*Mil (11)*	*Mil (11)*<br>*Mod (2)*	*Mil (10)*<br>*Mod (1)*	*Mil (11)*	*Mil (14)*<br>*Mod (2)*
Eye disorders							2 (1, 5.0%)
	Severity (n)						*Mil (1)*
Gastrointestinal disorders		16 (8, 61.5%)	9 (7, 58.3%)	14 (7, 58.3%)	8 (6, 50.0%)	21 (8, 66.7%)	25 (10, 50.0%)
	Severity (n)	*Mil (6)*<br>*Mod (5)*	*Mil (7)*	*Mil (6)*<br>*Mod (1)*	*Mil (6)*<br>*Mod (1)*	*Mil (8)*	*Mil (10)*
General disorders and administration site conditions		3 (3, 23.1%)	7 (6, 50.0%)	2 (2, 16.7%)	1 (1, 8.3%)	2 (2, 16.7%)	2 (2, 10.0%)
	Severity (n)	*Mil (3)*	*Mil (6)*	*Mil (2)*	*Mil (1)*	*Mil (2)*	*Mil (2)*
Immune system disorders		1 (1, 7.7%)	2 (2, 16.7%)				
	Severity (n)	*Mil (1)*	*Mil (2)*				
Infections and infestations					1 (1, 8.3%)	1 (1, 8.3%)	2 (2, 10.0%)
	Severity (n)				*Mil (1)*	*Mil (1)*	*Mil (2)*
Injury, poisoning and procedural complaints			2 (1, 8.3%)				2 (1, 5.0%)
	Severity (n)		*Mil (1)*				*Mod (1)*
Investigations		2 (2, 15.4%)		2 (2, 16.7%)			1 (1, 5.0%)
	Severity (n)	*Mil (2)*		*Mil (1)*<br>*Mod (1)*			*Mod (1)*
Musculoskeletal and connective tissue disorders		3 (3, 23.1%)	3 (3, 25.0%)	3 (3, 25.0%)	4 (3, 25.0%)	1 (1, 8.3%)	3 (3, 15.0%)
	Severity (n)	*Mil (3)*	*Mil (3)*	*Mil (3)*	*Mil (3)*	*Mil (1)*	*Mil (3)*
Nervous system disorders		5 (4, 30.8%)	3 (3, 25.0%)	9 (6, 50.0%)	3 (3, 25.0%)	4 (4, 33.3%)	7 (5, 25.0%)
	Severity (n)	*Mil (4)*	*Mil (3)*	*Mil (6)*	*Mil (3)*	*Mil (4)*	*Mil (5)*
Psychiatric disorders		1 (1, 7.7%)		1 (1, 8.3%)			1 (1, 5.0%)
	Severity (n)	*Mil (1)*		*Mil (1)*			*Mil (1)*
Renal and urinary disorders				1 (1, 8.3%)			
	Severity (n)			*Mil (1)*			
Respiratory, thoracic and mediastinal disorders		6 (5, 38.5%)	2 (2, 16.7%)	2 (2, 16.7%)	2 (2, 16.7%)	2 (2, 16.7%)	3 (3, 15.0%)
	Severity (n)	*Mil (5)*	*Mil (2)*	*Mil (1)*<br>*Mod (1)*	*Mil (2)*	*Mil (2)*	*Mil (3)*
Skin and subcutaneous tissue disorders			4 (4, 33.3%)	5 (4, 33.3%)	2 (2, 16.7%)	6 (5, 41.7%)	9 (6, 30.0%)
	Severity (n)		*Mil (4)*	*Mil (4)*	*Mil (2)*	*Mil (5)*	*Mil (6)*
Surgical and medical procedures		1 (1, 7.7%)					
	Severity (n)	*Mod (1)*					
Vascular disorders		1 (1, 7.7%)	1 (1, 8.3%)			1 (1, 8.3%)	
	Severity (n)	*Mil (1)*	*Mil (1)*			*Mil (1)*	
